# Supplementary material for: Zinc Supplementation, Inflammation, and Gut Integrity Markers in HIV Infection: A Randomized Placebo-Controlled Trial
Source: Nutrients. 2025 May 14;17(10):1671. doi: 10.3390/nu17101671 (PMC12114530; doi:10.3390/nu17101671)
Supplement: Supplementary file 1 [file nutrients-17-01671-s001.zip › nutrients-3633628-supplementary.pdf]

Adverse Events

	Placebo group n (%)	Treatment n (%)	Grade
**Clinical**			
Abdominal adhesions with intestinal torsion	0 (0.0%)	1 (0.4%)	2
Abcess post root canal	0 (0.0%)	1 (0.4%)	2
Bloating	0 (0.0%)	1 (0.4%)	1
Death	0 (0.0%)	1 (0.4%)	4
Diarrhea	0 (0.0%)	1 (0.4%)	1
Dizziness	0 (0.0%)	1 (0.4%)	2
High Blood Pressure	1 (0.4%)	0 (0.0%)	1
ER visit	0 (0.0%)	1 (0.4%)	3
Left groin hernia	0 (0.0%)	1 (0.4%)	1
Nausea	0 (0.0%)	8 (3.2%)	1
Pain in right hip and Sciatica	0 (0.0%)	1 (0.4%)	1
Peripheral Neuropathy	0 (0.0%)	1 (0.4%)	1
Scrotal Abcess	1 (0.4%)	0 (0.0%)	1
Stomach Pain	0 (0.0%)	2 (0.8%)	1
Worsening of depression	0 (0.0%)	1 (0.4%)	1
**Biological**			
High ALT and/or AST	1 (0.4%)	7 (2.8%)	1
High ALT and/or AST	0 (0.0%)	3 (1.2%)	2
High Cholesterol	11 (4.4%)	10 (4.0%)	1
High Cholesterol	2 (0.8%)	4 (1.6%)	2
High Cholesterol	0 (0.0%)	1 (0.4%)	3
High Creatine Kinase	1 (0.4%)	5 (2.0%)	1
High Creatine Kinase	0 (0.0%)	1 (0.4%)	3
High Creatinine	17 (6.8%)	22 (8.8%)	1
High Creatinine	1 (0.4%)	1 (0.4%)	2
High Creatinine	0 (0.0%)	1 (0.4%)	3
Low Hemoglobin	1 (0.4%)	2 (0.8%)	1
Low Phosphorus	2 (0.8%)	1 (0.4%)	1
Low Sodium	1 (0.4%)	2 (0.8%)	1
High Sodium	1 (0.4%)	0 (0.0%)	1
High Alkaline Phosphatase	0 (0.0%)	2 (0.8%)	1
High Bilirubin	4 (1.6%)	2 (0.8%)	1
High Bilirubin	1 (0.4%)	0 (0.0%)	2
High Glucose	15 (6.0%)	23 (9.2%)	1
High Glucose	3 (1.2%)	7 (2.8%)	2
High LDL	4 (1.6%)	5 (2.0%)	1
High LDL	4 (1.6%)	4 (1.6%)	2
High LDL	0 (0.0%)	1 (0.4%)	3
High Triglycerides	9 (3.6%)	22 (8.8%)	1

	High Triglycerides	2 (0.8%)	1 (0.4%)	2
	High Triglycerides	0 (0.0%)	1 (0.4%)	3
	High Triglycerides	0 (0.0%)	1 (0.4%)	4
	High Uric Acid	7 (2.8%)	11 (4.4%)	1
**Total**		89 (35.6%)	161 (64.4%)	
